# CLARITY: ChiLdhood Arthritis Risk Factor Identification STudY. Investigating the role of aberrant DNA methylation in juvenile idiopathic arthritis

**DOI:** 10.1186/1546-0096-9-S1-P275

**Published:** 2011-09-14

**Authors:** JA Ellis, R Chavez, L Gordon, AL Ponsonby, B Lim, J Akikusa, R Allen, R Saffery, J Craig, JE Munro

**Affiliations:** 1 Murdoch Childrens Research Institute , Melbourne, Australia; 2 Royal Children’s Hospital, Melbourne, Australia

## Background

Juvenile Idiopathic Arthritis (JIA) is a complex autoimmune disorder likely to be determined by multiple genetic and environmental factors. Mounting evidence shows that epigenetic variation influences autoimmune disease risk. The most well-studied epigenetic mark is DNA methylation; increased methylation of CpG dinucleotides can reduce gene expression. Nothing is currently known about the role of methylation in JIA. In 2008, we established CLARITY, a JIA Biobank that is collecting biospecimens and extensive information about environment from cases presenting to the Royal Children’s Hospital (RCH), Melbourne, Australia. A control sample of healthy children attending the RCH Day Surgery Unit has also been collected.

## Aims

To identify key differences in methylation patterns across the genome associated with the most common subtypes of JIA.

## Methods

DNA was isolated from peripheral blood mononuclear cell CD3+ CD4+ T cells obtained from incident oligoarticular (n = 10) and polyarticular (n = 4) JIA cases, and healthy age and sex matched controls (N = 14). Bisulfite converted DNA was applied to genome-wide methylation arrays (Illumina Infinium HumanMethylation27 Beadchips).

## Results

Methylation at ~27,000 CpGs across the genome was measured in cases and controls. Data was of high quality, with tight replication between technical replicates. Methylation was significantly different at 86 CpG sites between our cases and controls, following adjustment for false discovery rate (FDR). Ontology analyses revealed a significant bias towards immune system-related genes. Of particular interest was *LONRF2*, which has been associated with multiple autoimmune diseases, including rheumatoid arthritis*.* This gene showed a consistently increased degree of methylation in oligoarticular JIA cases when compared to controls. Consistent with these findings, work by others has demonstrated decreased LONRF2 expression in JIA.

## Conclusions

Our data suggests methylation is relevant to JIA disease risk. Further work will include in depth investigation of candidate genes such as *LONRF2*, concurrent gene expression analyses, and application of further samples to new-generation Infinium methylation arrays that measure over 450,000 CpG sites across the genome.

